# Design and development of a mobile-based self-care application for patients with depression and anxiety disorders

**DOI:** 10.1186/s12911-023-02308-y

**Published:** 2023-10-02

**Authors:** Khadijeh Moulaei, Kambiz Bahaadinbeigy, Esmat Mashoof, Fatemeh Dinari

**Affiliations:** 1 Department of Health Information Technology, Faculty of Paramedical, Ilam University of Medical Sciences, Ilam, Iran; 2 Medical Informatics Research Center, Institute for Futures Studies in Health, Kerman University of Medical Sciences, Kerman, Iran; 3 Department of Health Information Technology, Varastegan Institute for Medical Sciences, Mashhad, Iran

**Keywords:** Design, Mobile, Self-care, Application, Depression, Anxiety

## Abstract

**Background and Aim:**

Depression and anxiety can cause social, behavioral, occupational, and functional impairments if not controlled and managed. Mobile-based self-care applications can play an essential and effective role in controlling and reducing the effects of anxiety disorders and depression. The aim of this study was to design and develop a mobile-based self-care application for patients with depression and anxiety disorders with the goal of enhancing their mental health and overall well-being.

**Materials and methods:**

In this study we designed a mobile-based application for self -management of depression and anxiety disorders. In order to design this application, first the education- informational needs and capabilities were identified through a systematic review. Then, according to 20 patients with depression and anxiety, this education-informational needs and application capabilities were approved. In the next step, the application was designed.

**Results:**

In the first step, 80 education-information needs and capabilities were identified. Finally, in the second step, of 80 education- informational needs and capabilities, 68 needs and capabilities with a mean greater than and equal to 3.75 (75%) were considered in application design. Disease control and management, drug management, nutrition and diet management, recording clinical records, communicating with physicians and other patients, reminding appointments, how to improve lifestyle, quitting smoking and reducing alcohol consumption, educational content, sedation instructions, introducing health care centers for depression and anxiety treatment and recording activities, personal goals and habits in a diary were the most important features of this application.

**Conclusion:**

The designed application can encourage patients with depression and stress to perform self-care processes and access necessary information without searching the Internet.

**Supplementary Information:**

The online version contains supplementary material available at 10.1186/s12911-023-02308-y.

## Background

Depressive and anxiety disorders are significant contributors to worldwide disability [1] affecting up to 25% of general practice patients [2]. Normally, these disorders may not be as “brain disorders,“ but they do interfere with normal cognitive, emotional, and self-reflective functions [3]. Brain disorders include any conditions or disabilities that affect the brain [4, 5]. These disorders, caused by factors such as disease, genetics, or traumatic damage, encompass a range of conditions, including brain injuries, brain tumors, neurological diseases, as well as mental disorders like depression and anxiety [6, 7]. Depression and anxiety due to their nature always cause social, occupational and functional harm [8]. Studies have shown that if depression and anxiety are not treated, controlled and / or managed, they can lead to poor quality of life [9], increased risk of suicide [10, 11], job loss due to frequent absences [12], and premature mortality, persistent fatigue, sad and angry mood, decreased self-esteem and ability to perform daily activities, and increased risk of hospitalization [13]. On the other hand, due to the stigma associated with depressive and anxiety disorders, people are often reluctant to seek consultation and medication, which can hinder their access to effective psychological therapies [14]. One of the most effective ways to treat, control and / or manage these two disorders is self-care. Self-care as an independent factor can reduce the risk of disease complications [15].

Self-care processes help patients to control emotions, adhere to treatment, understand the treatment rationale, improve quality of life, reduce stress and anxiety, feel more secure, and increase life satisfaction. Also, these processes will ultimately maintain physical and mental health, reduce mortality, reduce health care costs, increase patient satisfaction and improve patients’ quality of life [16]. Mobile -based applications can be used as a platform for self-care services [17]. Mobile applications have become an all-encompassing tool for helping people to manage and control anxiety and depression symptoms [18], provide quick and easy access to health information, and improve interaction with therapists [19]. In other words, applications can aid people in managing their health, promoting a healthy lifestyle, and providing accurate information when and where it’s needed. Encouraging findings have been reported regarding the effectiveness of mobile-based applications for addressing depression and anxiety [20]. Lattie et al. [21] investigate the role of digital health interventions in improving depression and anxiety among students and concluded that applications are effective as computer, web, and virtual reality-based interventions in improving depression and anxiety. Almodovar et al. [22] also showed that mobile applications can increase self-confidence in coping skills and improve depressive and anxiety disorders.

To our knowledge, various studies have been done on the design and development of mobile apps to manage and control anxiety and depression. These applications have different capabilities, including patient monitoring, symptom tracking, emotional support, telecounseling, online training, medication reminders, BMI calculator, reporting and meditation management [23–25]. It should be noted that: none of these applications have all the features introduced in our study and only have some of these characteristics [23–25], for example, the 7 Cups of Tea application does not provide the possibility of interacting with the health care provider [26], and the language of these applications is not Farsi. Therefore, Iranian patients with stress and anxiety could not use these applications. Therefore, in the present study, we designed and developed a mobile-based self-care application for patients with depression and anxiety disorders. In this study, we answer the following three questions:

- What are the necessary capabilities and educational-informational needs of patients for designing a mobile-based self-care application through a literature review?
- What are the capabilities and educational-informational needs of patients for designing a mobile-based self-care application, considering the perspectives and opinions of patients with depression and anxiety?
- How is the application designed and what features does it have?

## Method

The present study is a developmental-applied study that was conducted in the following three stages.

### Stage 1: identify the capabilities and education- information needs of patients to design the application

According to various studies [17, 27–31], the first step in designing a mobile-based application is to identify information needs and necessary capabilities. These information needs and capabilities can be identified through a literature review [17, 29, 31], holding a panel of experts [28], focus groups with the end users [30], or interviewing target users [27]. In the first step of our study, patients’ information-educational needs and application capabilities were identified by literature review on January 1, 2022, from PubMed, Web of Science and Scopus databases. For this purpose, the following search strategy was used.

(Depression OR anxiety) AND (mobile-Based self-care application OR mobile-based Self-management application).

Inclusion criteria consisted of articles published in English, having access to the full text, and containing relevant information on the required information-educational needs and capabilities for designing the application. Exclusion criteria encompassed articles that did not provide clear information about self-care for anxiety and depression disorders through applications. The study excluded books, book chapters, letters to the editor, and conference abstracts.

**Fig. 1:**
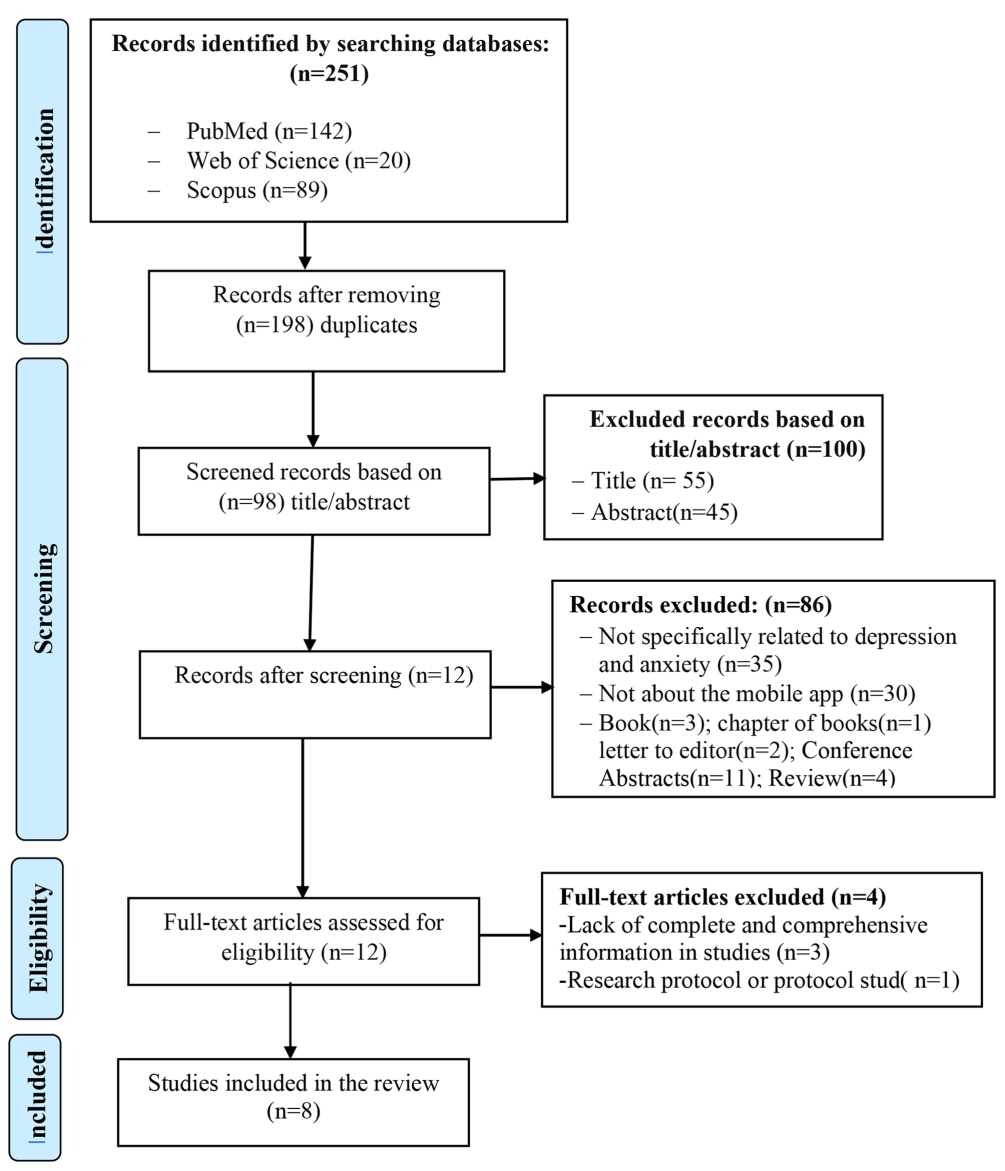
Selection of studies based on the PRISMA flowchart

Related articles were retrieved from the three introduced databases and entered into Endnote software. Two hundred and fifty-one articles were extracted from three databases: PubMed, Web of Science and Scopus. One hundred and forty-two studies from PubMed, 89 studies from Scopus and 20 study from Web of Science were retrieved. Four duplicate articles were excluded from the study. Then, 98 remaining sources were carefully examined and compared with inclusion and exclusion criteria. Then, the titles, abstracts and keywords of all articles were studied. Finally, 8 articles were included in the study (Fig. 1) [32–39]. We studied the full text of these articles and extracted the necessary data elements for designing and developing applications. Data collection was carried out using a data extraction form, and its validity was confirmed based on the opinions of two medical informatics and two psychiatric specialists.

### Stage 2: confirm the capabilities and education- informational needs to design the application

At this stage, the data collection tool was a questionnaire designed based on the educational information needs and capabilities identified in the previous stage. The questionnaire consisted of six parts, with the first part focusing on demographic information (4 questions). The second part: education-informational needs and capabilities in six parts: user profile (8 questions), clinical history (9 questions), lifestyle (14 questions), disease management and control (28 questions), sedation instructions (10 questions), and application capabilities (16 questions). Also, for each part of the questionnaire, an open-ended question was mentioned under the heading “Other cases”. The Content Validity Ratio (CVR) was employed to assess the questionnaire’s content validity. Two medical informatics and three psychiatric specialists completed the questionnaire to calculate the CVR. These people had the experience of conducting various researches in the field of anxiety and stress and collaborating in the design of self-care applications. In order to calculate the CVR, the expert panel was instructed to rate each question using a three-point scale: “essential,“ “helpful but not essential,“ and “not essential” [17, 40]. Afterward, the CVR was determined utilizing the subsequent formula:

$$CVR = \frac{n_{e-N/2}}{N/2}$$

#### *Note

n represents the count of experts choosing the “essential” option, while N represents the total number of experts.

As per Lawshe’s criteria for CVR, when the expert panel consists of five members, the minimum acceptable value for each item is 0.99 [40]. In this research, the minimum acceptable CVR value for each question, as determined by the experts, was 1.00. Additionally, the overall CVR ratio was computed as 1.00.

Moreover, the reliability of the questionnaire was evaluated by Cronbach’s alpha and was confirmed with a value of 0.902 (Appendix A). Sampling was not performed at this stage, and all patients with depression and anxiety (40 patients) referred to the Hamzeh Medical Center affiliated to Fasa University of Medical Sciences (Fasa city, Iran) from December 2021-December 2022 were included in the study. It should be noted that during this period of time, 510 patients with psychiatric disorders had referred to this center, 40 of them were suffering from depression and anxiety. In order to participate, an invitation was sent to all of these patients. Thirty people accepted the invitation and finally 20 people entered the study according to the inclusion criteria. The inclusion criteria were:

- At least 18 years old.
- Having a smart mobile phone literacy.
- Declare informed consent to participate in the study.
- Do not suffer from acute cognitive and mental disorders except depression and anxiety.

The questionnaire was electronically designed, and its link was sent to patients on January 12, 2022. All questionnaires were completed by January 20. It is worth mentioning that to incentivize participation, each participant received a gift card worth 1,000,000 Iranian Rials for a local grocery store in Fasa city.

The results obtained from the questioner were analyzed by SPSS 23.0. The answers “completely unnecessary”, “unnecessary”, “neutral”, “necessary”, and “completely necessary” with scores from 1 to 5 was given. Also, descriptive statistics (frequency, mean, and standard deviation (SD) were used. In accordance with the opinion of the research team and several psychiatrists, information-educational needs and application capabilities with a mean greater than and equal to 3.75 (75%) were considered to design and develop the application. A cut-off score of 3.75 or higher indicates that only items rated as “necessary”, and “completely necessary” by patients are included in the application design. Other studies [17, 31, 41] related to application design showed that by considering mean greater than and equal to 3.75 (75%) as a cutoff, more important and necessary information-educational needs and capabilities will be selected for application design. As a result, the application will be more efficient and useful.

### Stage 3: design and development a prototype of the mobile-based application

At this stage, based on the education-informational needs and capabilities approved in the previous stage, the prototype was designed with the Java programming language in an Android Studio programming environment. SQLite DB was used to design the database. After entering the information and saving it by the patients, the mobile application sends the information to the application database. After the information is saved, they can access stored information, edit it or add new information. Finally, patients can report the information stored in PDF format and send the report to their physicians via social networks or email. During the study, the use of the application was free for patients. Moreover, it should be noted that we did not design a user interface for physicians, and the patient’s communication with physicians will be through social networks and email.

Given the popularity of the Android operating system in Iran, the prototype of this application was specifically designed for Android OS version 4.4 KitKat and higher. Notably, both the application and its database were developed by a Mobile App Design company, ensuring that only the patient can access and share the information stored in the application’s database with their therapist.

### Ethical considerations

The code of ethics with the number IR.KMU.REC.1399.025 was obtained from the ethics committee of Kerman University of Medical Sciences on March 18, 2020. Patients’ informed consent was obtained before participating in the study. The participation of physicians and patients in the study was also completely voluntary and it was possible for them to leave the study at any time.

## Results

### Stage one: identify the education- informational needs and capabilities to design the application

An overview of selected studies is presented in Table 1. Moreover, Fig. 1 shows the search results and the study selection process.

**Table 1:** An overview of selected studies is presented in Table 1

Ref	Study aims	Study type	Number of participants	Information-educational needs and capabilities	Study results
[42]	Investigating the relationship between the severity of depression and the latent trait of interest in the schematic self-referential processing of cases of depressive symptoms using a mobile application	Quasi-experimental	70 (36 males and 34 females)	Recording the depressive symptoms, self-care training, and patient’s follow-up by therapists	- Excellent built-in compatibility of the K-CESD-R mobile application - High treatment adherence rate for all participants - High follow-up rate for most of participants
[33]	Identifying the implications of smartphone apps for the control and management of depression and anxiety	Not mentioned	14 (5 males and 9 females)	Recording the depressive symptoms, automated prompts and reminders, planning for doing more exercise meditating more and drinking less alcohol, user authentication or approval, the audio tracks, and lifestyle management (how to exercise, reduce alcohol consumption and do meditation activities)	- Immediate symptomatic alleviation - Individual empowerment - Interpersonal support - promoting reductionist biomedical conceptualizations of mental ill health by mobile apps
[43]	Evaluating the effectiveness of a web- and mobile-based intervention on treatment adherence	Randomized Controlled Trial (RCT)	164 (40 males and 164 females)	Interactive sessions and intervention sessions include text, testimonials, exercises, and audio and video clips, audio sequences introduce relaxation exercises, reminders, and online-based assessment patients	- Statistically significant between-group difference in Quick Inventory of Depressive Symptomatology (QIDS) scores at posttreatment in favor of the intervention group - Significant improvement in favour of the intervention group for secondary outcomes such as quality of life, anxiety, and insomnia severity
[44]	Investigating the effect of psychoeducational interventions on anxiety and self-esteem of women with breast cancer using mobile applications and online support groups	RCT	82 women	Educational materials (including texts, animations, images, quizzes, audio files, and video clips, video clips for demonstrating how to accurately execute the exercises), proper diet management, stress management (addressing topics such as stress complications and anxiety symptoms, teaching the techniques of stress management and emotion management, thought stopping, diaphragmatic and conscious breathing, guided imagery, and progressive muscle relaxation), self-esteem and anger management (addressing anger management methods and problem-solving and including keys to the image gallery, aims, about us, and references)	- Significant reductions in the scores of anxiety and its two subscales (state anxiety and trait anxiety) - Increasing in the postintervention mean scores of self-esteems in the intervention group
[45]	Investigating the effectiveness of an internet-based intervention and a mobile phone-based application for students with high stress	RCT	150 (38 males and 112 females)	Trainings such as rumination and worrying, time management, procrastination, test anxiety, sleep, motivation, nutrition and exercise, and dealing with writer’s block and concentration, diary for recording different states of mood, the possibility of uploading images for the therapist, the possibility of reporting files in pdf format and completing evaluation forms related to psychological tests, automatic daily messages containing short motivational prompts, and ultrabrief training exercises via SMS (short message service)	- Reducing consequences of college-related stress and depression by mobile apps
[46]	Investigating the feasibility and usability of a mobile-based interactive chatbot program in reducing attention deficit symptoms	RCT	46 (20 males and 26 females)	Providing various training related to attention deficit, such as the cause, symptoms, and treatment—specifically, medications, Usage time and user log patterns are recorded for the analysis, daily assessment of the user’s concentration, mood, and state of anxiety, recording behavior change, emotional control, and mindfulness, time management, impulsivity, depression, and anxiety, and medication reminders Double-click to accept corrections and edit your text	- Significant reduction of attention deficit symptoms - Improvement in the Attention-Deficit/Hyperactivity Disorder (ADHD) symptoms
[47]	Evaluation of the treatment outcomes of people with depression using three self-guided mobile apps	RCT	348 (118 males and 266 females)	Recording daily changes in mood, video game, provided daily health tips for overcoming depressed mood such as self-care (e.g., taking a shower) or physical activity (e.g., taking a walk), providing suggestions for mindfulness and behavioral exercises, reminders, and providing psychotherapy evaluation forms	- Improvement depressive symptoms - Closing the treatment gap for underserved communities by mHealth
[48]	Identification and analysis of current and future evidence of applications, social media, chatbots and virtual reality	Not mentioned		Capturing longitudinal, dense and multimodal mental health data for use in diagnosis and monitoring, connections to clinical care, and remote patient monitoring.	- Better management and control of major depression; anxiety, bipolar and psychotic disorders by mobile apps

### Stage 2: confirm the capabilities and education- informational needs to design the application

Table 2 shows the demographic information of patient’s participant in stage two of the study. The majority of participants (60%) were female. Most age groups were 31–40 years old. Also, the majority of participants (80%) were suffering from depression and anxiety.

**Table 2:** Participants Demographic Information

Variable	Variable types	Frequency (Percent)
Gender	Men	8(40) 12(60)
	Women	
Age	18–30	8(40) 10(50) 2(10)
	31–40	
	>=41	
Education level	Diploma	6(30) 7(35) 6(30) 1(5)
	Bachelor	
	Master	
	PhD	
Disorders type	Depression	2(10) 2(10) 16(80)
	Anxiety	
	Depression and Anxiety	

Findings related to education-informational needs and capabilities required for application design included six categories include: user profiles, clinical records, lifestyle, disease management and control, relaxation instructions, and application capabilities (Table 3). The importance of each of these education-informational needs and capabilities is presented in Table 3. Of 80 education-informational needs and capabilities, 68 education-informational needs and capabilities with a larger mean and equal to 3.75 (75%) were considered for application design.

In the user profile, national code, age, weight, education, address and contact number with a mean of less than 3.75 were not included in the application design. In the lifestyle category, underlying diseases and in the application capabilities category, BMI calculation, lectures, relaxing music and games and intellectual puzzles were excluded from the study and were not considered for designing the application.

**Table 3:** Information-educational needs and capabilities for application design

Category	Education-informational needs and capabilities	Mean(± SD)	Decision (necessary or unnecessary)
User profile	First name & last name	3.81 (± 1.51)	√
	National code	3.40(± 0.14)	×
	Age	3.12 (± 1.08)	×
	Weight	3.74(± 1.32)	×
	Height	3.81(± 1.24)	√
	Education level	3.27(± 1.14)	×
	Address	3.31(± 1.11)	×
	Contact number	3.51(± 0.25)	×
Clinical history	Underlying disease	3.40(± 1.14)	×
	Family history of mental disorder and type of disorder	3.85(± 1.04)	√
	Duration of the disorder	3.80(± 1.15)	√
	Suicide history	3.95(± 0.75)	√
	Blood group	3.90(± 1.55)	√
	Hospital history	3.90(± 1.11)	√
	The first hospitalization	3.80(± 1.43)	√
	Number of hospitalizations	3.75(± 1.43)	×
	History of smoking and alcohol	3.80(± 1.23)	√
Life style	Sport	4.25(± 1.33)	√
	Sleep management	4.20(± 1.15)	√
	Nutrition	4.15(± 0.93)	√
	Proper weight	3.75(± 0.91)	√
	Smoking and drinking alcohol	3.75(± 1.08)	√
	Stress and anxiety Management	4.20(± 1.43)	√
	Existence of bad habits	4.35(± 1.04)	√
	Overcoming to wrong beliefs	3.85(± 1.18)	√
	Overcome to failures	3.95(± 1.14)	√
	Personal hygiene	3.75(± 0.91)	√
	Physical activity	4.05(± 1.19)	√
	Strengthen the mind and body	3.95(± 1.14)	√
	Healthy sex	4.10(± 1.16)	√
	Social support and healthy relationships	4.25(± 1.07)	√
Disease management and control	Introduction of anxiety and depression disorders	4.10(± 1.02)	√
	Symptoms of anxiety and depression disorders	4.05(± 0.94)	√
	Complications of anxiety and depression disorders	4.10(± 1.37)	√
	Deep relaxation exercises	4.05(± 1.19)	√
	Prevent the aggravation of the effects of anxiety and depression disorders	3.95(± 1.14)	√
	Overcoming stress and negative thoughts	4.10(± 1.16)	√
	Being Optimistic	4.05(± 0.94)	√
	Anger management	4.20(± 1.19)	√
	Smoking and drinking alcohol	4.05(± 1.31)	√
	Drug use and Addiction	4.20(± 1.05)	√
	Dealing with worry	4.05(± 1.31)	√
	Manage conflict at work, school or in relationships	4.10(± 1.37)	√
	Proper communication with others	4.00(± 1.29)	√
	Anxiety and nervous attacks	4.10(± 1.21)	√
	How to get away from stressful relationships and environments	4.07(± 1.04)	√
	Health nutrition and diet	4.05(± 1.09)	√
	How to maintain mental health	3.95(± 1.29)	√
	Increasing the self confidence	4.10(± 1.21)	√
	Easing fear	4.05(± 1.43)	√
	High focus	3.95(± 1.27)	√
	Positive communication and social interactions	4.00(± 1.07)	√
	Make a better sense on yourself	3.95(± 1.19)	√
	Motivation for more activity	4.00(± 1.17)	√
	Reduce restlessness	4.10(± 1.07)	√
	Self-care	4.00(± 1.29)	√
	Hopeful sentences	4.10(± 1.21)	√
	Daily programming	4.15(± 1.04)	√
Relaxation instructions	Slowly and regularly breathe	4.05(± 1.09)	√
	Strengthen muscles	3.90(± 1.21)	√
	Prayer	3.75(± 1.37)	√
	Music therapy	4.05(± 1.14)	√
	Aromatherapy	4.05(± 1.09)	√
	Mental imagery	3.75(± 1.16)	√
	Mindfulness	3.75(± 1.20)	√
	Meditation	3.85(± 1.13)	√
	Walking with mindfulness or yoga	3.80(± 1.39)	√
	Repeat soothing words	3.75(± 1.30)	√
Application capabilities	Calculate BMI	3.50(± 1.16)	×
	Lectures	3.30(± 0.92)	×
	Provide clinical history	3.70(± 1.38)	√
	Introducing counseling centers to receive health services	3.85(± 1.30)	√
	Management of medications	3.85(± 1.34)	√
	Management of nutrition and diet	4.10(± 1.21)	√
	Notebook	3.80(± 1.36)	√
	Communication with doctors, consultants and other patients	3.75(± 1.27)	√
	Appointment reminder	4.05(± 1.09)	√
	Relaxing music	3.75(± 1.31)	√
	Games and intellectual puzzles	3.65(± 1.38)	×
	Application settings (such as font, size and color of content)	3.60(± 3.25)	×

*Note: ×: Unnecessary and **√**: Necessary

### Stage 3: design and development a prototype of the mobile-based application

**Fig. 2:**
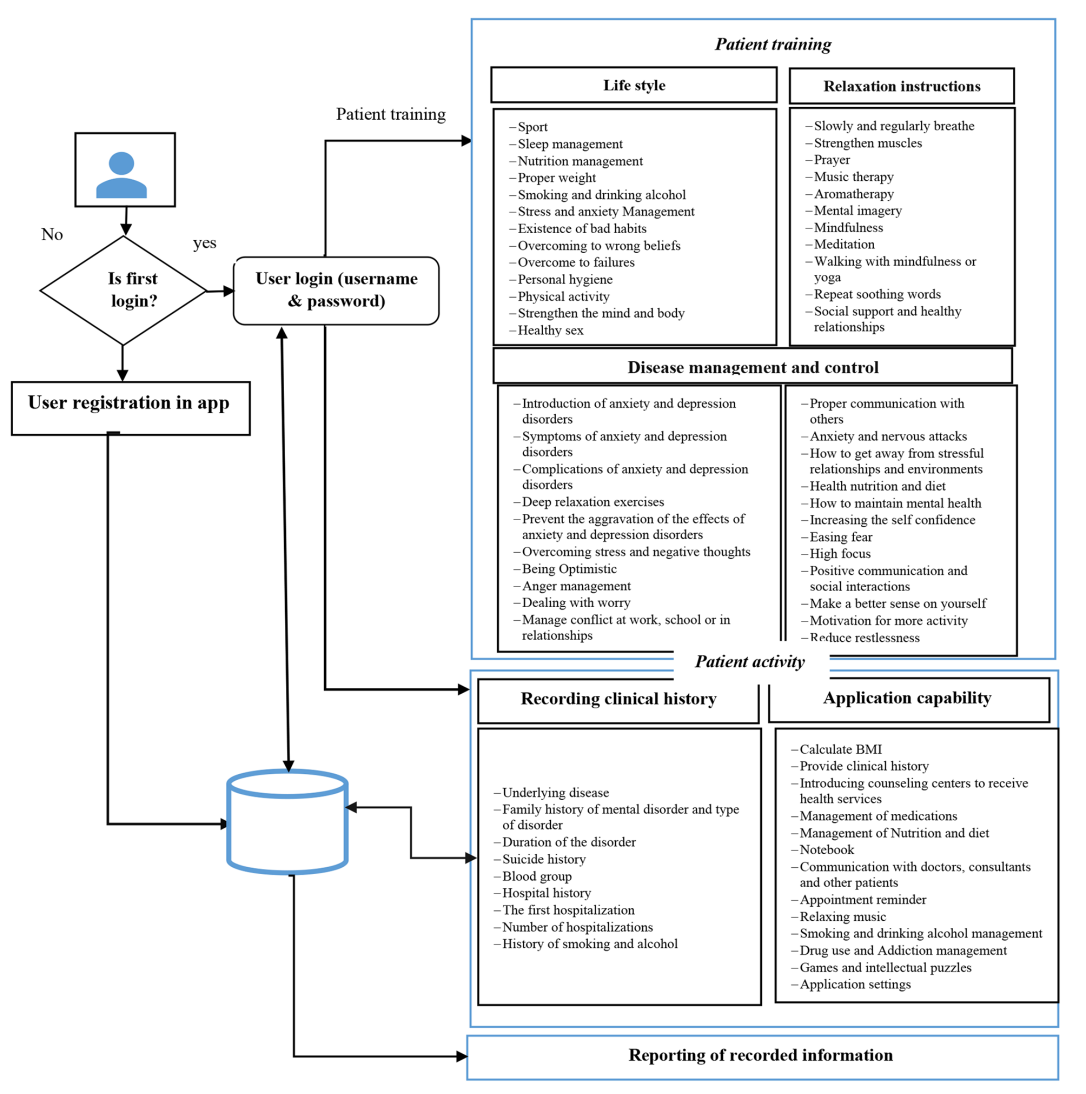
The architecture of the designed mobile self-care application

According to the results obtained in the needs assessment stage, a mobile-based self-care application for patients with anxiety and stress disorders was designed with the Java programming language in the Android Studio environment. The architecture of this self -care app is shown in Fig. 2.

**Fig. 3:**
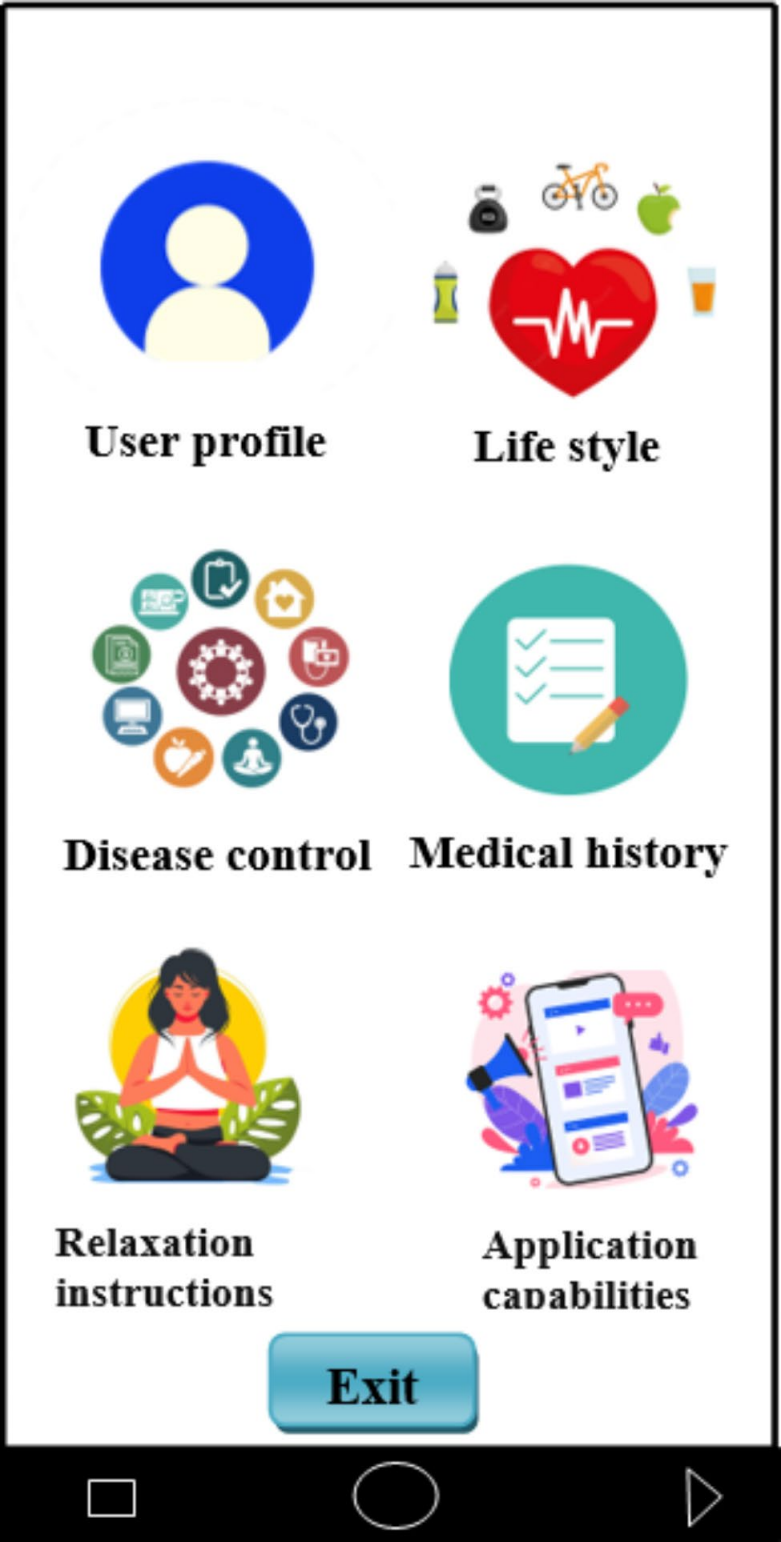
Home page of depression and anxiety self-care application

This application has six main sections namely user profiles, clinical records, lifestyle, disease management and control, relaxation instructions, and application capabilities on the main page of the application (Fig. 3). By clicking on each of the icons of these sections, a subset of their related features will be displayed. In total, this application has 20 pages for features of each section: user profiles (1 page), clinical records (2 page), lifestyle (3 page), disease management and control (10 page), relaxation instructions (2 page), and application capabilities (2 page). In the following, each of these sections is described.

In the user profile section, the patient can register after entering the application and by entering a username and password enter the application.

**Fig. 4:**
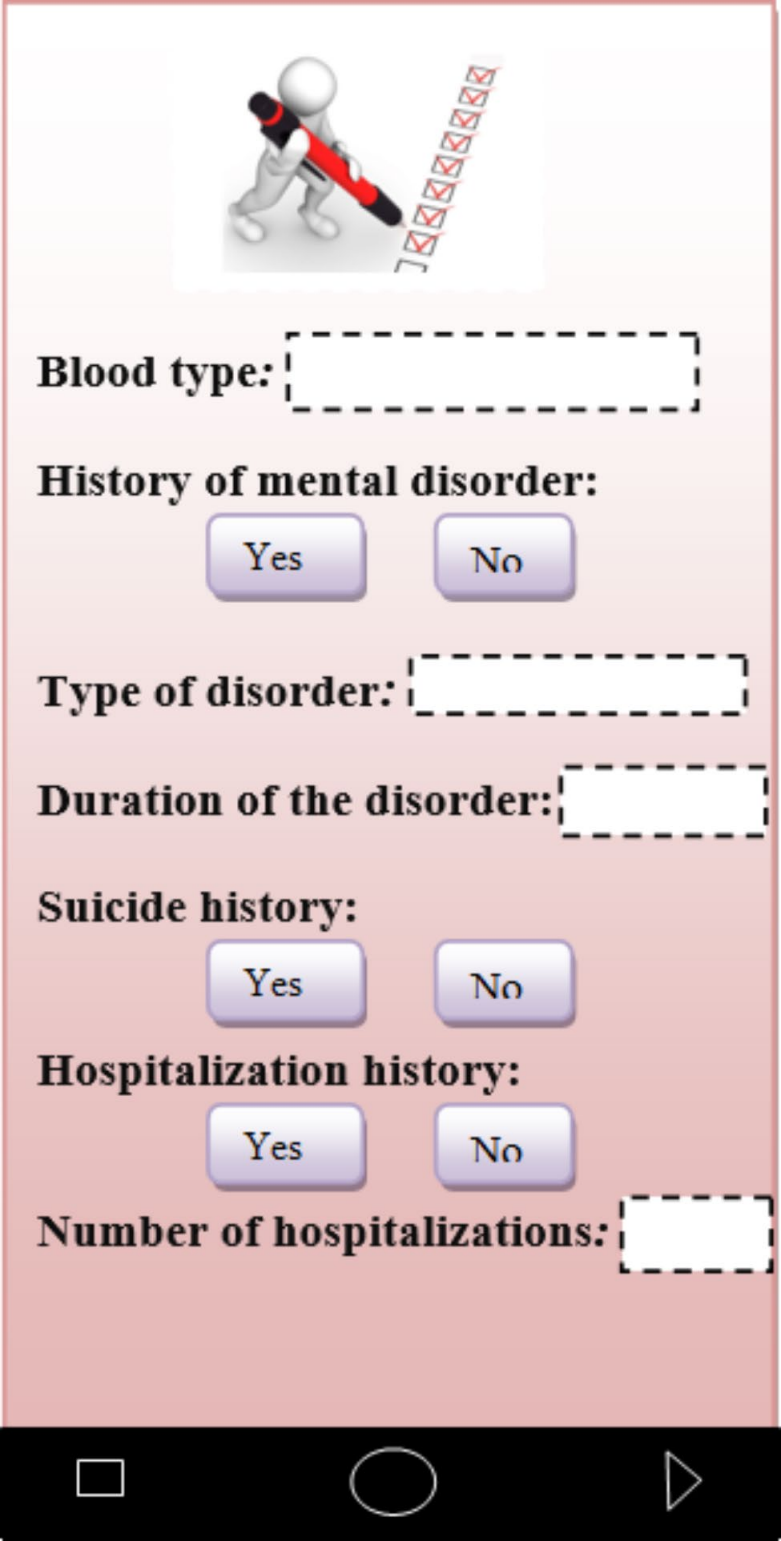
Recording of medical and clinical records

In the clinical history category, the patients can save various information about blood group, family history of mental disorder and type of disorder, duration of the disorder, history of suicide, history of hospitalization, time of first hospitalization, number of hospitalizations and history of smoking and alcohol consumption on their mobile phone and send them to his/her doctor as a pdf file (Fig. 4).

In the lifestyle category, educational information in the form of videos and texts related to exercise, sleep, proper nutrition, proper weight, smoking and alcohol, stress and anxiety management, healthy bad habits, how to overcome wrong beliefs, how to overcome failures, personal health, physical activity, mind and body strengthening, healthy sex, social support and healthy relationships are provided.

**Fig. 5:**
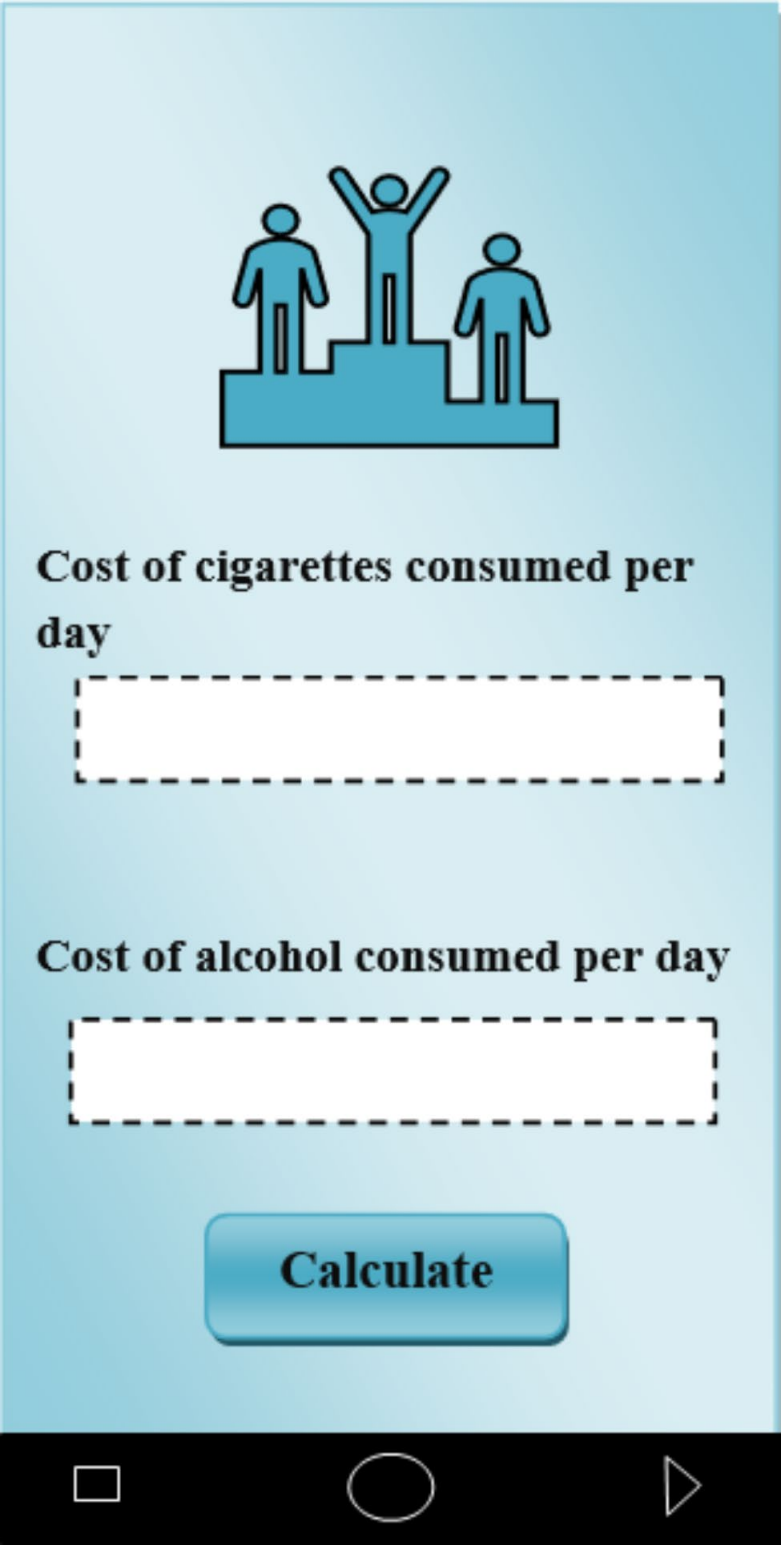
Quitting drinking and smoking

In the disease management and control category, the complications caused by depression and anxiety can be controlled and managed. As an example, part of this application is intended for quitting smoking and alcohol. The patient can enter the days he does not smoke or drink alcohol in the application. Also, enter the cost of cigarettes and alcohol consumed per day and number of cigarettes smoked daily in the application. Then, by clicking on “calculate”, the app tells the patient how much money you have saved by not buying cigarettes so far, as well as how many days you have been clean and how many cigarettes you have not smoked so far. Seeing statistics can give patients positive energy and make it easier to quit smoking or drinking (Fig. 5). Moreover, in order to get rid of addiction and drugs, patients can send their current history to their therapists on a daily basis through social networks in the form of text, audio, video or PDF files. Then, the therapists will provide them with the necessary guidance and recommendations.

In the category of relaxation instructions, different methods of relaxing the patient through slow and regular breathing, muscle strengthening, prayer, music therapy, aromatherapy, mental imagery, mindfulness, meditation, walking with mindfulness or yoga and repetition of soothing words is taught. These trainings were provided to the patient in the form of text, videos and voice.

It should be noted that the educational material featured in our application, which includes topics such as lifestyle guidance, relaxation instructions, and disease management and control, was meticulously curated from the websites of the Iranian Psychological Association (https://iranpa.org/) and Iranian Psychiatrist Association (http://www.psychiatrist.ir/main/). To ensure the accuracy and alignment of this content with recommended best practices in treatment, a rigorous review process was undertaken. Specifically, the content underwent evaluation and approval by two experienced psychiatrists who possess expertise in the field of mental health and have a deep understanding of evidence-based treatment approaches. This collaborative effort between medical professionals and our development team aimed to ensure that the educational content within our application adheres to the highest standards of quality and reliability, ultimately providing users with valuable and trustworthy information to support their mental health and well-being.

**Fig. 6:**
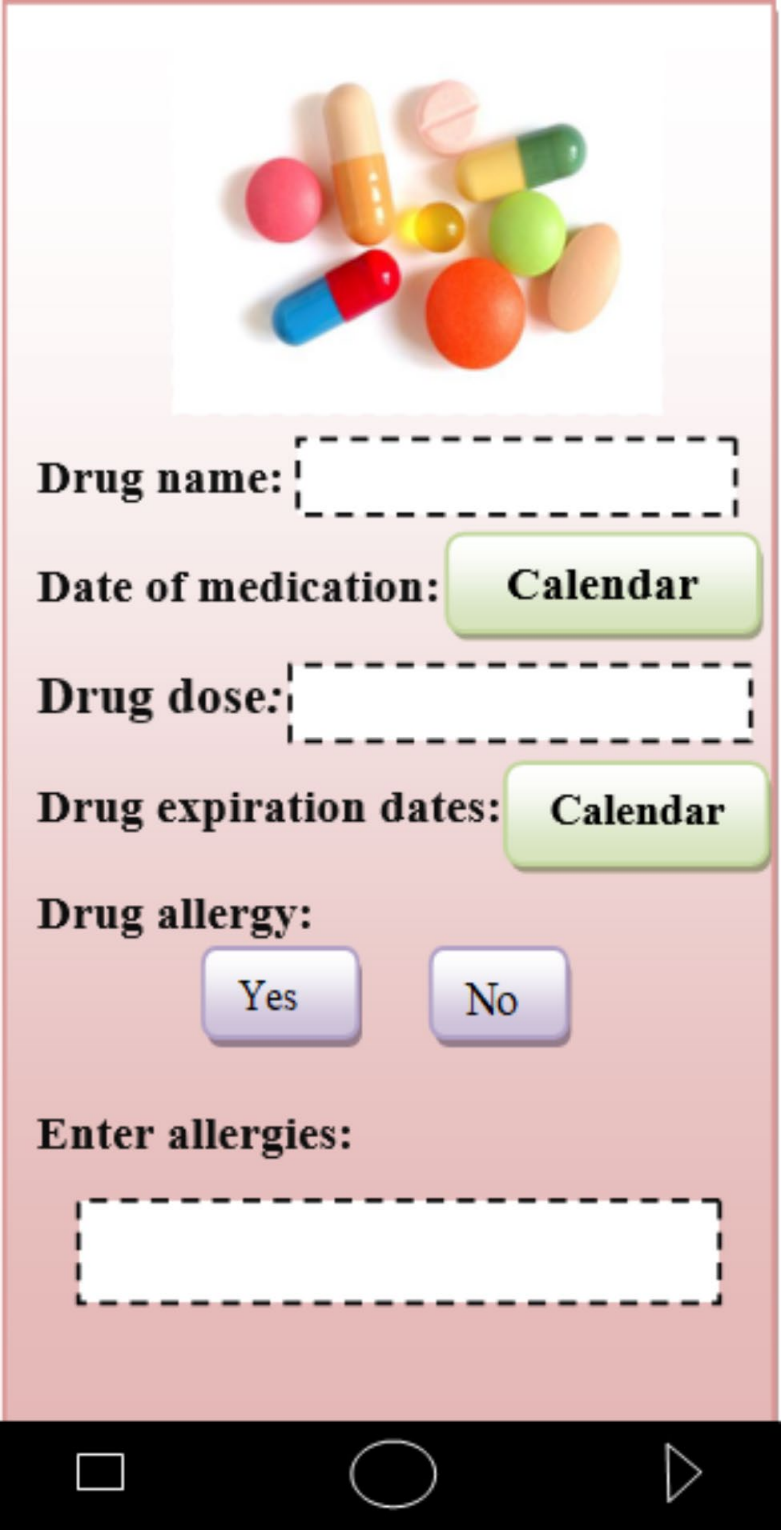
Drug management

In the capabilities category, addresses and phone numbers of medical centers in Fars province (Iran) were introduced to patients to receive counseling services. Patients could contact these centers to get an appointment or go to these centers in person according to the addresses provided. In the field of drug management, nutrition and diet management, patients could set a diet plan for themselves. For example, in the drug management section, patients could enter the drug name, dosage, drug allergies, and drug use date. According to the time and date of use, the necessary reminders were given to the patient (Fig. 6). In the notebook section, patients can write down information about their mental health, relationships, mood or feelings. Also, record her/his activities, personal goals or habits.

In the section of communication with doctors, consultants and other patients, a group was formed on WhatsApp and Telegram, patients could talk to doctors and consultants and other patients and share their experiences in this groups. Also, they could ask their questions. In the section of appointment reminder, patients could enter the time and date of appointment, doctor’s name and office address. Like other applications, patients can customize reminders based on physicians’ recommendations. For example, the patient needs to be advised by the doctor to take a medicine every day at 8 am, the patient can take his medicine on time by set a reminder for every day at 8 am. Based on the recorded time and date, reminders are provided to the patient automatically. It should be noted that reminders can act as guidance or messages to help facilitate behavior change and increase adherence to medication or treatment and patient attendance at appointments [49]. Moreover, reminders can reduce the need to memorize, reduce the number of missed drug doses, reduce treatment interruptions, avoid forgetting to take medications, and perform laboratory tests on time [49, 50].

In the application settings, the user can change settings such as font and size, font color and themes.

**Fig. 7:**
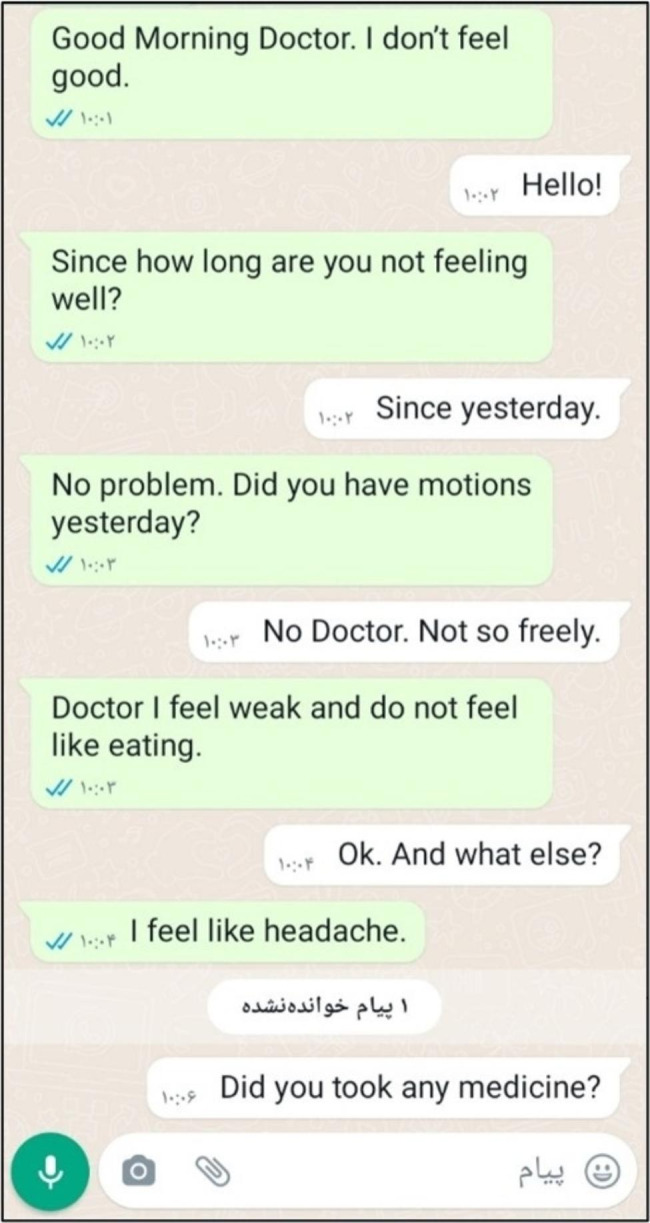
An example of a conversation between a patient and a therapist

It should be noted that after registering information in the application, patients can report them in PDF format and send them to their therapists via email or social networks. Patients could also talk to their therapist through social networks. Figure 7 shows an example of conversations between the patient and the therapist.

**Fig. 8:**
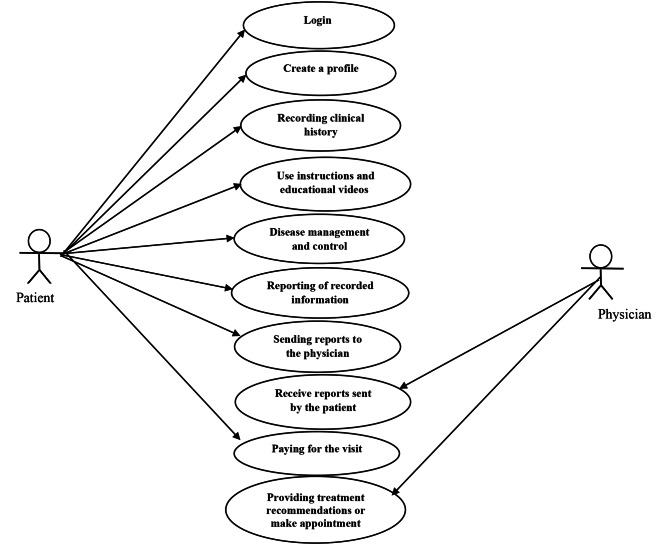
Use Case diagram for patient and physician

In order to better understand the capabilities of the designed application, we designed a use-case diagram for patients and physicians. The application allows patients to: (1) log into the system, (2) Create a profile, (3) record their clinical history, (4) View tutorials with self-care instructions, (5) Using the app’s capabilities to manage and control the disease, (6) reporting on recorded clinical information, (7) sending reports to physicians through social networks or email, and (8) paying for the visit (Fig. 8). All patient data is stored in the application database. Moreover, the application allows physicians to: (1) receive reports sent by the patient, and (2) provide treatment recommendations or make an appointment (Fig. 8).

## Discussion

In this study, a mobile-based self-care application was designed and developed for patients with depression and anxiety disorders. The designed application allows the patient to register and enter through a username and password and record their clinical history in PDF format and send it to the doctor. Also, this app can help to improve patients’ lifestyles by providing educational information on reducing and controlling anxiety and depression in the form of videos, text and voice. Moreover, management of medications dose and time of use, the ability to record activities, personal goals and habits in a diary, the introduction of depression and anxiety treatment centers, communication with other patients and doctors were other features of this application. Wasil R et al. [51] reviewed applications were designed for depressive and anxiety disorders in a review study. The most common features used in these applications included educational and self-assessment services to patients, how to gain calm, concentration and meditation. Also, in our study, educational services were provided to improve self-care processes and how to achieve relaxation, concentration and meditation. Instructions for concentration and relaxation let person to get rid of internal and external factors that bother him/her. These instructions can help people to return to a normal state and perform daily routine activities in the present [52] and reduce stress and anxiety in people with depressive and anxiety disorders [53–55].

Fuller-Tyszkiewicz et al. [56] also designed a self-monitoring application with name BlueWatch to improve the well-being of adults with depressive symptoms. This app is organized based on the principles of Cognitive Behavioral Therapy (CBT) in six modules of psychological education about depression and an introduction to CBT, behavioral activation, cognitive reconstruction, problem-solving skills, assertiveness, and treatment methods to prevent Recurrence of disease. Blue Watch features also included short audio education activities, daily practice and self-monitoring functions (using daily mood recordings), short welcome video, training with the app and dashboard (to store patient activities and texts). The present study provides daily exercises in the form of relaxation instructions in the designed application. Patients by performing daily exercises such as calm and regular breathing, muscle strengthening, prayer, music therapy, aromatherapy, mental imagery, mindfulness, meditation, walking with mindfulness or yoga, repeating soothing words help themselves to reduce stress and anxiety.

Management of smoking, shisha, alcohol and drugs was another feature of the application designed in our study. Deady et al. [57] also were considered a section for managing of smoking, hookahs, alcohol, and drugs in their application, along with other information-educational needs and capabilities such as training programs (prevention of exacerbation of effects of anxiety and depression disorders, overcoming stress and negative thoughts, how to get away from relationships and stressful environments) relaxation instruction, sleep management, physical activity and exercise, and daily programming. Other studies [58–60] have shown that there is a direct link between depression and anxiety and smoking. They can increase the severity of anxiety and depression in these patients over the time. So, in self-care applications for these patients, it is better to allocate a section for smoking, shisha, alcohol management.

Patient management of medications was another feature of the application designed in the present study. This feature can help patients to enter the name of the drug, dosage, drug allergies and drug use date. In order to take the medicine, the necessary warnings were given to the patient according to the time and date of use. Philip Kaare Løventoft et al. [61] designed an application called life management to support patients with depression. This application has various capabilities for user registration, measuring the patient’s depression based on the WHO Major Depression Inventory (MDI) questionnaire, Mood, appetite and sleep registration, calendar and event types, location tracking and mapping (providing data on patient movement patterns for Predicting phases of depression) and routine management (to help users with daily tasks such as getting out of bed, taking a shower, and daily programming). Also, it had capabilities to record a list of drugs that could be edited by the user, reminding the use of drugs in the Medication management section.

In evaluating a mobile application, there are always problems, advantages and disadvantages, which will be analyzed in the following. Furthermore, Wei and et al. [62], underscored the significance of an interactive process that didn’t bewilder users or require numerous iterations for comprehension, as such hurdles hindered their sustained engagement with the application. For example, offering clear explanations of how the mHealth intervention operated, including guidance on what steps to take next, encouraged ongoing usage.

The unwillingness of patients [63, 64] to cooperate in the evaluation process is one of the major issues with evaluating mobile applications. Patients’ lack of knowledge and awareness of the advantages and uses of these applications, as well as a lack of sufficient evidence regarding the effectiveness of anxiety and stress applications, may also contribute to their unwillingness to cooperate. Therefore, ways to encourage patient cooperation should be offered. One of these solutions is to give patients adequate information about the utility and efficacy of the application. The application’s adoption and use, as well as collaboration, can all be enhanced by this solution. Additionally, inviting patients from different races, ethnicities, genders, ages, and education statuses to a meeting of the research team to discuss this application and its advantages can be helpful [65]. If patients are made aware that self-care tools may aid in illness management and control. Then, it will be simpler for patients to embrace these apps since they would think that by following self-care the applications, their recovery will be substantially accelerated [64, 66, 67]. The team can highlight the advantages of an anxiety and depression self-care app, like better health information access‌ [68], lower medical errors and treatment costs, improved coordination among healthcare providers, and reduced patient travel [69]. They can also inform patients that the app offers greater flexibility, enabling them to spend less time at treatment centers and more time on daily tasks [70].

Privacy concerns during patient evaluations are another issue that has been identified in prior research [71–73]. Designers of applications should strive to keep patient information private. Therefore, each patient must have a unique username and password for self-care applications. In addition, the research team should provide sufficient assurance to the patients that the information they enter will remain confidential while using the application. The ease of use of self-care applications is another patient concern [74, 75]. This issue can be resolved by providing patients with the necessary training in the form of multiple training sessions, as well as by preparing educational files in the form of video and text regarding the use of the application for patients and doctors [24].

Another issue in mobile application evaluation is the availability of various evaluation tools (questionnaires such as mobile app rating scale (MARS) and system usability scale (SUS), heuristic evaluation, think aloud, etc.) and the lack of flexibility of these tools. For instance, Zhou et al. [76], argued that the SUS questionnaire, when applied to aspects unique to mobile apps, fails to yield the specific information required for evaluating mobile applications effectively, highlighting the need for tailored evaluation tools in the mobile app domain. To solve this issue, the primary objective of each research’s evaluation should be identified, and then the right tool should be chosen. The tool selected for evaluation should focus on various dimensions related to evaluation quality, readability and cultural sensitivity of content, usability and features of health applications [77]. Another drawback of application evaluation studies is the length of time needed to complete the evaluation. A mobile application may initially appeal to the patient and the therapist in a way that yields a positive initial evaluation result, but over time, the outcome changes. As a result, it is preferable to evaluate over time. It should be noted that imbalances in access to online health care systems that are a reflection of well-known socioeconomic disparities in access to online services. The same factor makes using mobile devices for remote service delivery to rely on patients who have more facilities and skills and may unjustly burden those who are less able with treatment using newer technologies [78]. One of the difficulties that evaluators encounter when assessing applications for anxiety and stress management programs is this disparity. In this case, researchers may decide to exclude study participants who lack smart phones, internet access, adequate bandwidth, a sufficient level of literacy, or the desire to take part in the study.

App evaluation can also have benefits. Different aspects of an application are examined in different ways during evaluations. For instance, the following three factors are taken into account and scrutinized during the usability evaluation: (1) Having greater usability, (2) more user satisfaction (meets the user’s expectations), and (3) easier learning (the operation can be learned very quickly by observation). Or, ten indicators are highlighted in Nielsen’s assessment: (1) display Visibility of system status, (2) consistency and standards, (3) user control and freedom, (4) error prevention, (5) recognition rather than recall, 6) flexibility and efficiency of use, (7) flexibility and efficiency of use, (8) aesthetic and minimalist design, (9) honesty in expressing mistakes and providing solutions - assisting users in identifying, analyzing, and resolving errors; and (10) assistance and documentation [79, 80]. The design team will identify and address any issues with these dimensions after evaluating the application. A user-friendly application will subsequently be created for users. However, once all the issues are resolved, the patients’ continued use of the application will increase. Patients will be less satisfied and use these applications less if an application is not usable or does not have the necessary quality for the patient’s goals [81]. According to some studies [82, 83], users will be dissatisfied with the application if there are potential delays in their response to the application, a lack of optimal speed for the information and content it contains, difficulty in learning and comprehending its features. So, the amount of use of the application with them decreases day by day.

However, by assessing applications, it is possible to learn how well they work to enhance self-care practices, self-management, self-efficacy, control over a disease, and disease recovery [84–87]. Patients may utilize an application continually if it is efficient in the dimensions that were presented. Organizations and hospitals may also encourage people to utilize these resources. Patients who make use of these effective tools will reduce the number of people who visit medical facilities physically, preventing overcrowding. Additionally, patients will spend less time traveling to treatment facilities and pay lower treatment costs [17]. On the other hand, studies on app evaluation that publish their findings can assist other app designers and developers in creating the best possible apps. For instance, a city or village’s culture may not support the use of a particular color in the design of an application. When creating their applications, designers are not permitted to use this color. On the other hand, these people can spend less time and money designing and developing an application after seeing the results of these studies. One of the additional benefits of evaluation is that it raises patients’ knowledge and awareness of applications of self-care in the field of health [88–90]. Patients can then easily learn how to use the applications and become familiar with the various features that a self-care application should have. The evaluation of applications also increases the likelihood that patients will develop loyalty and a sense of community [90]. The patient will feel more accountable for enhancing the application’s quality when he participates in its evaluation and will offer the research team the necessary feedback.

Evaluations may have disadvantages in addition to their benefits. The expenses incurred to motivate individuals to participate in the evaluation process are the first disadvantages of evaluation. For instance, patients typically decline offers of free participation in studies. So, researchers must pay them the required fees to take part in the study. On the other hand, researchers may need to buy tools to record the evaluation process in order to evaluate an application according to the type of evaluation method, such as video cameras, microphones and headsets, audio recording tools, evaluation analysis software, etc. [91]. On the other hand, it takes a lot of time to complete evaluation process. In order to assess an application’s long-term effectiveness, users occasionally need to use it for days and hours. As a result, both the research team and the patients will find it boring.

Sometimes, in some evaluation methods, the evaluation of an application for users does not produce satisfactory and good results [82, 83]. Because of this, users of this application might become discouraged and stop using it altogether. These situations can occasionally arise from a lack of time for an evaluation or from selecting an improper evaluation technique. Therefore, care must be taken in selecting the method and length of the evaluation in accordance with the purpose of the designed application. It should be noted that one of the disadvantages of self-care applications is that they are constantly being evaluated because of updates. These ongoing assessments could be very expensive for designers and the people who develop them.

### Limitations

In the needs assessment stage in order to confirm the capabilities and educational-informational needs necessary for designing the application, we included only 20 patients in the study. Moreover, Patients’ education-informational needs and application capabilities required to design the application were identified only in accordance with the opinions of patients referred to Hamzeh in Fasa speciality and sub-speciality clinics and were not used viewpoint of psychologists and psychiatrists. It is suggested to include more patients in the needs assessment stage in future studies, and also to use the opinions of psychologists and psychiatrists. Also, in this study, the usability of the designed application were not evaluated and its effects on improving and reducing anxiety and stress were not considered. In another study, the usability and effects of app on improving and reducing anxiety and stress will investigated. Through a Randomized Controlled Trials (RCT) study, the effects of the app on improving and reducing anxiety and stress can be investigated.

## Conclusion

In the present study, a mobile-based self-care application for patient with depression and anxiety disorders was designed and developed. The designed application provides mechanisms to collect and store patients’ information and send them to physicians. In addition, patients can actively and dynamically participate in self-care processes with the continuous use of this application, and access to required information without search in the Internet. Also, this app has great potential for situations where patients cannot see their doctor in person, such as during the COVID-19 pandemic.

### Electronic supplementary material

Below is the link to the electronic supplementary material.

Supplementary Material 1

## Data Availability

The datasets used and/or analyzed during the current study are available from the corresponding author on reasonable request.

## Abbreviations

HIS: Hospital Information System
EPN: Electronic Progress Note
