# Supplementary material for: Design and development of a mobile-based self-care application for patients with depression and anxiety disorders
Source: BMC Med Inform Decis Mak. 2023 Oct 2;23:199. doi: 10.1186/s12911-023-02308-y (PMC10544565; doi:10.1186/s12911-023-02308-y)
Supplement: Supplementary file 1 — Supplementary Material 1 [file 12911_2023_2308_MOESM1_ESM.docx]

**Identify the capabilities and education- information needs to design and development a mobile-based self-care application for patient with depression and anxiety disorders**

**Section A:** Demographic and clinical characteristics

1. What is your education level?

- Men
- Women

1. What is your age in years?

- 31-30
- 31-40
- >=41

1. What is your education level?

- Diploma
- Bachelor
- Master
- PhD

1. What is your type of disorder?

- Depression
- Anxiety
- Depression and Anxiety

**Section B:** Information-educational needs and capabilities for application design and development

| **Category** | **Education-informational needs and capabilities** | **Completely unnecessary** | **Unnecessary** | **Neutral** | **Necessary** | **Completely necessary** |
| --- | --- | --- | --- | --- | --- | --- |
| **User profile** | First name & last name |  |  |  |  |  |
|  | National code |  |  |  |  |  |
|  | Age |  |  |  |  |  |
|  | Weight |  |  |  |  |  |
|  | Height |  |  |  |  |  |
|  | Education level |  |  |  |  |  |
|  | Address |  |  |  |  |  |
|  | Contact number |  |  |  |  |  |
| **Clinical history** | Underlying disease |  |  |  |  |  |
|  | Family history of mental disorder and type of disorder |  |  |  |  |  |
|  | Duration of the disorder |  |  |  |  |  |
|  | Suicide history |  |  |  |  |  |
|  | Blood group |  |  |  |  |  |
|  | Hospital history |  |  |  |  |  |
|  | The first hospitalization |  |  |  |  |  |
|  | Number of hospitalization |  |  |  |  |  |
|  | History of smoking and alcohol |  |  |  |  |  |
| **life style** | Sport |  |  |  |  |  |
|  | Sleep management |  |  |  |  |  |
|  | Nutrition |  |  |  |  |  |
|  | Proper weight |  |  |  |  |  |
|  | Smoking and drinking alcohol |  |  |  |  |  |
|  | Stress and anxiety Management |  |  |  |  |  |
|  | Existence of bad habits |  |  |  |  |  |
|  | Overcoming to wrong beliefs |  |  |  |  |  |
|  | Overcome to failures |  |  |  |  |  |
|  | Personal hygiene |  |  |  |  |  |
|  | Physical activity |  |  |  |  |  |
|  | Strengthen the mind and body |  |  |  |  |  |
|  | Healthy sex |  |  |  |  |  |
|  | Social support and healthy relationships |  |  |  |  |  |
| **Disease management and control** | Introduction of anxiety and depression disorders |  |  |  |  |  |
|  | Symptoms of anxiety and depression disorders |  |  |  |  |  |
|  | Complications of anxiety and depression disorders |  |  |  |  |  |
|  | Deep relaxation exercises |  |  |  |  |  |
|  | Prevent the aggravation of the effects of anxiety and depression disorders |  |  |  |  |  |
|  | Overcoming stress and negative thoughts |  |  |  |  |  |
|  | Being Optimistic |  |  |  |  |  |
|  | Anger management |  |  |  |  |  |
|  | Dealing with worry |  |  |  |  |  |
|  | Manage conflict at work, school or in relationships |  |  |  |  |  |
|  | Smoking and drinking alcohol |  |  |  |  |  |
|  | Drug Use and Addiction |  |  |  |  |  |
|  | Proper communication with others |  |  |  |  |  |
|  | Anxiety and nervous attacks |  |  |  |  |  |
|  | How to get away from stressful relationships and environments |  |  |  |  |  |
|  | Health nutrition and diet |  |  |  |  |  |
|  | How to maintain mental health |  |  |  |  |  |
|  | Increasing the self confidence |  |  |  |  |  |
|  | Easing fear |  |  |  |  |  |
|  | High focus |  |  |  |  |  |
|  | Positive communication and social interactions |  |  |  |  |  |
|  | Make a better sense on yourself |  |  |  |  |  |
|  | Motivation for more activity |  |  |  |  |  |
|  | Reduce restlessness |  |  |  |  |  |
|  | Self-care |  |  |  |  |  |
|  | Hopeful sentences |  |  |  |  |  |
|  | Daily programming |  |  |  |  |  |
| **Relaxation instructions** | Slowly and regularly breathe |  |  |  |  |  |
|  | Strengthen muscles |  |  |  |  |  |
|  | Prayer |  |  |  |  |  |
|  | Music therapy |  |  |  |  |  |
|  | Aromatherapy |  |  |  |  |  |
|  | Mental imagery |  |  |  |  |  |
|  | Mindfulness |  |  |  |  |  |
|  | Meditation |  |  |  |  |  |
|  | Walking with mindfulness or yoga |  |  |  |  |  |
|  | Repeat soothing words |  |  |  |  |  |
| **Application capabilities** | Calculate BMI |  |  |  |  |  |
|  | Lectures |  |  |  |  |  |
|  | Provide clinical history |  |  |  |  |  |
|  | Introducing counseling centers to receive health services |  |  |  |  |  |
|  | Management of medications |  |  |  |  |  |
|  | Management of Nutrition and diet |  |  |  |  |  |
|  | Notebook |  |  |  |  |  |
|  | Communication with doctors, consultants and other patients |  |  |  |  |  |
|  | Appointment reminder |  |  |  |  |  |
|  | Relaxing music |  |  |  |  |  |
|  | Games and intellectual puzzles |  |  |  |  |  |
|  | Application settings (such as font, size and color of content) |  |  |  |  |  |
